# Molecular characterization of histidine and tyrosine decarboxylating *Enterococcus* species isolated from some milk products

**DOI:** 10.1186/s12866-025-03940-6

**Published:** 2025-04-23

**Authors:** Obeid Shanab, Faten Fareed, Ahmed Y. Nassar, Hanan H. Abd-Elhafeez, Ahmed Shaban Ahmed, Mona A. El-Zamkan

**Affiliations:** 1 Department of Biochemistry, Faculty of Veterinary Medicine, South Valley University, Qena, 83523 Egypt; 2 Department of Biochemistry, Faculty of Medicine, Assiut University, Assiut, 71526 Egypt; 3 Department of Cell and Tissues, Faculty of Veterinary Medicine, Assiut University, Assiut, 71526 Egypt; 4 Department of Food Hygiene and Control (Milk Hygiene), Faculty of Veterinary Medicine, South Valley University, Qena, 83523 Egypt

**Keywords:** Biogenic amines, Dairy products, *Enterococcus* spp., Decarboxylase activity, Antimicrobial resistance, Biofilm, Virulence genes

## Abstract

**Background:**

Fermented foods can cause adverse effects on human health because of the biogenic amines (BAs) accumulating through amino acid decarboxylation. This study investigated the presence of BAs including tyramine and histamine in 240 samples of some cheese and fermented milk samples using high-performance liquid chromatography. Another aim of this study is to isolate and identify *Enterococcus* spp. as the most important and frequent BA producer in the examined samples. The isolated *Enterococcus* spp. was investigated phenotypically for their capacity to produce amino acid decarboxylase enzyme using decarboxylase microplate assay, and genotypically through molecular detection of some genes encoding amino acid decarboxylation (*tyrdc* and *hdc*). Biogenic amines producing enterococci were then investigated for their antimicrobial resistance, biofilm production as well as their virulence determinants.

**Results:**

Tyramine and histamine could be detected in 86.7 and 87.9% of the investigated samples with 52.9% being contaminated with *Enterococcus* spp. Significant correlation between the incidence of *Enterococci enterococci* and BAs formed in the examined samples (*P* < 0.0001). *tyrdc* and *hdc* genes were detected in 85 and 5% of amino acid decarboxylating *Enterococcus* spp., respectively. A high percentage of *Enterococcus* isolates (57.5%) were multidrug-resistant and resistance against penicillin was widespread among isolates followed by tetracycline, vancomycin, erythromycin and linezolid. Also, 77.5% of the isolates were capable of forming biofilms and a highly significant correlation (*P* < 0.0001) was found between biofilm formation and multidrug resistance. The results showed that the rates of most virulence genes *gelE*, *esp*, *ace*, *asa1*, and *cylA* were 77.5. 47.5, 47.5, 35 and 7.5%, respectively, while the *hyl* gene was not detected in any isolates.

**Conclusion:**

The study highlights the significant presence of BAs (TYM and HIS) in cheese and fermented milk samples, with a strong correlation between enterococci contamination and TYM production. The high prevalence of tyramine-producing *Enterococcus* species poses a notable public health concern especially with the high prevalence of multidrug-resistant, biofilm production and virulence in BAs producing *Enterococcus* spp. in dairy products, emphasizing the urgent need for improved antimicrobial stewardship among food producers and veterinarians to mitigate the risk of transferring resistant strains to humans.

**Supplementary Information:**

The online version contains supplementary material available at 10.1186/s12866-025-03940-6.

## Introduction

Biogenic amines (BAs) are small nitrogenous compounds characterized by a low molecular weight and a chemical structure that includes one or more amino groups (NH_2_). These compounds exhibit biological activity within human bodies [1]. BAs are formed through enzymatic decarboxylation of free amino acids or amination and transamination of aldehydes and ketones [2]. HIS, TYM, putrescine, cadaverine, tryptamine, spermidine, spermine, and phenylethylamine are the most significant BAs found in certain foods and beverages, produced by the decarboxylation of their corresponding amino acids [1].

Biogenic amines have been extensively studied as components of food that have the potential to cause harmful toxic reactions and intoxications that are detrimental to human health [3]. The European Food Safety Authority (EFSA) has stated that “HIS and TYM are considered as the most toxic and food safety relevant, and fermented foods are of particular BA concern due to associated intensive microbial activity and potential for BA formation.”, being HIS is associated with “scombroid fish poisoning” while TYM is linked to the “cheese reaction” [2, 4]. Moreover, the combined cytotoxic effects of HIS and TYM in food can potentially worsen adverse effects such as headaches, hypertension, and gastrointestinal issues, causing synergistic toxicity and leading to more severe symptoms than when present individually. Even at non-toxic concentrations (below EFSA’s safety threshold), histamine enhances the cytotoxicity of tyramine at levels commonly found in food [5].

The study of BAs in food is a crucial research area, with around 535 articles published in the last year alone based on a search in the PubMed database. Research on BAs is carried out for three main reasons: toxicological implications, as the ingestion of food rich in these compounds can pose risks to consumers; their potential role as indicators of food quality; and their physiological effects on human health [2].

Biogenic amines are indicators of food quality because their formation results from the microbial decarboxylation of precursor amino acids. Various microorganisms such as *Enterococcus*,* Enterobacteriaceae*, *Pseudomonas* spp., *Micrococcus* and Lactic Acid Bacteria (LAB) have been identified to possess amino acid decarboxylase activity. These organisms, which are capable of producing BAs, may either be naturally present in the raw materials microbiota or introduced during different stages of food processing [6]. Among these, the genus *Enterococcus* stands out as a particularly efficient producer of BAs, particularly TYM, which is associated with the phenomenon known as the “Cheese reaction” [7]. Enteroocci are recognized for their significant presence in the natural microbiota of many artisanal cheeses and have been found to outcompete other bacteria such as lactobacilli and lactococci, thereby influencing the overall quality of dairy products [c^8^].

Certain *Enterococcus* species are opportunistic pathogens and have the potential to cause infections in humans, especially in healthcare settings [9]. Hence, the existence of these species in food can represent a risk to human health [10]. The pathogenicity of *E. faecalis* and *E. faecium*, is primarily contributed to the presence of particular virulence factors including cytolysin (*cylA*), gelatinase (*gelE*), enterococcal surface protein (*esp*), collagen adhesion (*ace*), hyaluronidase (*hyl*) and aggregation substances (*asa1*) [11]. These factors are essential in the bacteria’s virulence mechanisms, particularly in aiding biofilm formation during the colonization of host tissues [12].

The issue of antibiotic resistance, particularly multi-resistance, poses a significant challenge to public health in the context of *Enterococcus* infections because of the potential for treatment failure, particularly in immunocompromised individuals [13]. *E. faecium* and *E. faecalis* have been found to exhibit inherent resistance to multiple classes of antibiotics, including aminoglycosides, cephalosporins, polymyxins, lincomycin, and clindamycin, as a result of genetic alterations that provide a fundamental resistance against these antibiotics [14]. Furthermore, certain strains of *Enterococcus* have developed resistance to β-lactams, glycopeptides, higher doses of aminoglycosides, and linezolid through the acquisition of resistance genes [15]. The emergence of vancomycin-resistant enterococci (VRE) has been recognized as a “high priority” and “serious threat” by the World Health Organization [16] and the U.S. Centre for Disease Control and Prevention [17]. Clinical isolates of *E. faecalis* and *E. faecium* are resistant to commonly used antibiotics, including ampicillin and vancomycin, in cases of bacteraemia, endocarditis, and urinary and pelvic infections. Additionally, there is a growing concern about the emergence of resistance to last-resort antibiotics, such as linezolid, among enterococcal strains [18].

This study’s objectives were assessing the hazardous presence of tyramine (TYM) and histamine (HIS) BAs in cheese and fermented milk samples through their detection using high-performance liquid chromatography (HPLC), considering their permissible limits and acceptable daily intakes (ADI) for humans of different ages. It also explored the role of *Enterococcus* species in biogenic amine formation by isolating and identifying *Enterococcus* spp. from the samples, followed by phenotypic and genotypic detection of histidine and tyrosine amino acid decarboxylase activity. Additionally, the study aimed to determine the antimicrobial susceptibility profile, biofilm production, and virulence determinants of the histidine and tyrosine amino acid decarboxylating *Enterococcus* spp.

## Materials and methods

### Collection of samples

A total of 240 random samples of various dairy products, including Romy (Ras) Cheese, Cheddar Cheese, Labn Rayeb (Fermented Milk; both small-scale and large-scale), and Yoghurt (both small-scale and large-scale) (40 samples each), were collected from different dairy shops, dairy cattle rural houses, street vendors, and supermarkets in Qena city, Egypt.

### Biogenic amine determination

#### Extraction and derivatization of biogenic amine

The determination of BAs was conducted through acid extraction and derivatization [19]. All the chemicals and reagents utilized in this particular procedure were obtained from Sigma-aldrich. The homogenization of two grams of each sample was carried out in 20 mL of 0.1 M HCl (7647010) with the addition of 100 mg L-1 of internal standard (1,7-diaminoheptane; D17408). Subsequently, the mixture was centrifuged at 1400 g for 20 min at 4 °C, and the resulting supernatant was collected. Following this, an additional extraction was performed using 20 mL of 0.1 M HCl. The combined acid extracts were then adjusted to a volume of 50 mL with 0.1 M HCl and filtered through Whatman™ Grade 54 Quantitative Filter Paper.

To derivatize the samples, 0.5 mL of each acid extract was mixed with 150 mL of a saturated NaHCO3 solution (25080094) and the pH was brought to 11.5 using around 150 mL of 1.0 M NaOH (1310732). Subsequently, 2 mL of dansyl chloride (HY-D0017) in acetone (67641) solution (10 mg mL-1 dansyl chloride/ acetone), was added to the alkaline amine extract. The resulting mixture was then placed in an incubator at 40 °C for 60 min. Any remaining dansyl chloride was eliminated by the addition of 200 mL of 300 g L-1 ammonia solution. After a 30-minute incubation at 20 °C shielded from light, each sample was adjusted to 5 mL with acetonitrile (75058) and filtered through a 0.22 mm PTFE filter.

#### HPLC analysis

High-performance liquid chromatography (HPLC) was employed to analyze the prepared samples, with the parameters detailed in Table S1. The estimation BAs followed the methods of Martuscelli et al. [19] and Magwamba et al. [20], with minor adjustments. The mobile phase comprised HPLC-grade acetonitrile (eluent A; 75058) and ultrapure water (eluent B), both filtered and degassed prior to use. Chromatographic separation was achieved using an isocratic elution method with a 60:40 ratio of acetonitrile to ultrapure water. The flow rate was set at 1.2 ml/min for 6.5 min to ensure complete separation. Derivatized amines were detected using a UV detector at 254 nm. A set of biogenic amine standards, including histamine dihydrochloride (99%; 53300) and tyramine hydrochloride (98%; T2879), and their mixtures were analyzed alongside the test samples. During analysis, standard solutions were periodically injected between test samples to ensure chromatographic consistency. Each sample was injected twice. The peak heights of the biogenic amine standards were used to generate standard curves, enabling the determination of amine concentrations in the test samples by comparing peak area retention times to the standard curves. The results of the biogenic amines were then compared to the permitted limits set by global organizations.

#### Estimated daily and weekly intakes (EDI and EWI) of the samples examined

The human daily and weekly intakes of BAs from the examined samples were estimated using the following equation [21]. Furthermore, the results were compared to the Acceptable Intake levels established by EFSA [4].

$${\rm{EDI = }}\frac{{Mean\:BA\:conc{\rm{entration}} \times \:Average\:food\:consumption}}{{Body\:weight}}$$

### Role of *Enterococcus* species in biogenic amine formation in the examined samples

#### Isolation of *Enterococcus spp.* from the samples examined

From each dairy sample, 25 g was mixed with 225 ml of Brain Heart Infusion (BHI). Subsequently, 10 µL of the homogenized mixture was added into BHI broth (Oxoid, CM1135) and incubated for 24 h at 37˚C. Following this, the samples were streaked onto Bile Aesculin Azide Agar plates (Oxoid, CM0888) and further incubated for 24 h at 37˚C [22]. The appearance of dark brown or black colouration around the colony was provisionally considered indicative of *Enterococcus* spp., which was later identified on the species level using the VITEK 2 System (Version 08.01, bioMe´rieux, USA) and confirmed by PCR.

#### Phenotypic and genotypic detection of amino acid decarboxylase activity of isolated *Enterococcus* spp.

The investigation of *Enterococcus* isolates for HIS and TYM amino acid decarboxylation was conducted using Decarboxylase media. The procedure involved utilizing Low Nitrogen Decarboxylase Broth (LND) and Low Glucose Decarboxylase Broth (LGD) in a 96-well Microtiter plate, following the method of Espinosa-Pesqueira et al. [6]. LND broth was prepared to reduce false positive results, while LGD broth was designed to minimize false negative reactions by high fermentative bacteria. Both broth media were supplemented with a mixture of the precursor amino acids L-Histidine monohydrochloride (Sigma-Aldrich) and L-Tyrosine disodium salt (Sigma-Aldrich), before the test.

A fresh 24-hour colony of each *Enterococcus* isolate was suspended in 0.85% NaCl physiological solution until it reached a turbidity of approximately 0.5 on the McFarland standard. Aliquots of amino acid broth (200 µL) and 20 µL saline solution (decarboxylase control assay: DCA) were added to the wells of a 96-well microtiter plate. For the negative decarboxylase assay (NDA), 200 µL of broth without amino acids and 20 µL of bacterial suspension were added to the wells. For the positive decarboxylase assay (PDA), 200 µL of amino acid broth and 20 µL of each bacterial suspension were added to the wells. Then the microplates were incubated at 30 °C for 24 h. In the PDA wells, positive outcomes were indicated by the appearance of a purple coloration, which signifies increased alkalinity. While positive outcomes in LGD broth were confirmed when no colour change was observed in the wells of PDA, while a yellow colour appeared in the NDA wells, indicating significant acidification resulting from bacterial growth. Negative results were determined when there was no change in the PDA wells’ colour or when a purple colour emerged in the wells of NDA due to alkaline compounds apart from BAs. Then BAs producing *Enterococcus* isolates on decarboxylase media were submitted to molecular detection of corresponding genes.

### Antimicrobial resistance of obtained *Enterococcus* isolates

The antimicrobial susceptibility of the isolated TYM and HIS decarboxylating *Enterococcus* isolates was conducted using the disc diffusion method. The antibiotics tested included ampicillin (AMP, 10 µg), penicillin (P, 10 units), vancomycin (VA, 30 µg), erythromycin (E, 15 µg), tetracycline (TET, 30 µg), ciprofloxacin (CIP, 5 µg), chloramphenicol (CHL, 30 µg), linezolid (LZD, 30 µg), gentamicin (CN, 10 µg), kanamycin (K, 30 µg), and nitrofurantoin (F, 300 µg). According to the guidelines of the Clinical Laboratory Standards Institute [23], the diameters of the inhibitory zones were measured and classified as sensitive or resistant.

#### Determination of the MAR index

The MAR index was calculated using the method outlined by El-Zamkan et al. [24], where the number of antibiotics an isolate is resistant to (a) is divided by the total number of antibiotics tested (b). The calculation formula is shown below:

MAR index= $$\:\raisebox{1ex}{$a$}\!\left/\:\!\raisebox{-1ex}{$b$}\right.$$

### Biofilm formation

Biofilm production was evaluated using the method described by El-Zamkan and Mohamed [22] with microtiter plates (MTP). Each overnight-grown isolate in Tryptic Soy Broth (TSB) with 1% glucose at 37 °C was transported (20 µL) into three wells of sterile 96-well polystyrene microtiter plates containing 180 µL of fresh TSB with 1% glucose, then incubated for 24 h at 37 °C. Wells with 200 µL of uninoculated TSB served as negative controls. After incubation, wells were drained, washed three times with 300 µL phosphate-buffered saline, fixed with methanol for 20 min, stained with 2% crystal violet for 15 min, washed twice with distilled water, and air-dried. The dyed adherent cells were resolubilized in 150 µL of 33% acetic acid for 30 min. The optical density (OD) of each well was measured at 570 nm using a microtiter plate reader. The cut-off value (ODc) is calculated as the average negative control OD plus three times the standard deviation of the negative control. Based on this, the Enterococcus isolates were classified as follows: non-biofilm producers if OD is less than ODc, weak biofilm producers if OD is between ODc and 2ODc, moderate biofilm producers if OD is between 2ODc and 4ODc, and strong biofilm producers if OD is greater than 4ODc, as described by Stepanović et al. [25].

### Detection of tyrosine and histidine decarboxylation and virulence encoding genes in isolated *Enterococcus spp.*

The QIAamp DNA Mini kit (Qiagen GmbH, Hilden, Germany) was employed to extract DNA from enterococci cultures (applied biotechnology, ABT001, Korean). The extracted DNA from all *Enterococcus* isoltes was used for molecular confirmation of isolated *E. faecium *and *E. faecalis*, utilizing the 16 S rRNA [26] and *atpA* genes [27], respectively. Additionally, the DNA was screened for the presence of tyrosine-decarboxylase (*tyrdc*) and histidine-decarboxylase (*hdc*) genes [28], as well as the virulence genes *cylA*, *gelE*, *esp*, *ace*, *hyl*, and *asa1* [29, 30]. PCR amplification for confirmation and virulence genes was performed in a 25- µL mixture reaction that contained the EmeraldAmp Max PCR Master Mix (Takara, Japan) (12.5 µL), 1 µL of each primer, water (5.5 µL) and DNA template (5 µL). While for screening the presence of tyrosine-decarboxylase (*tyrdc*) and histidine-decarboxylase (*hdc*) gene fragments in *Enterococcus* isolates, PCR reaction contained 25 µl EmeraldAmp Max PCR Master Mix (Takara, Japan), 2 µl of each primer, 10 µl DNA template and nuclease-free water to a final volume of 50 µl. An Applied biosystem 2720 thermal cycler was used to perform the reaction, and electrophoresis on agarose gel (1.5%) (Applichem, Germany, GmbH) separated the PCR amplicons. All the primers are displayed in Table S2.

### Statistical analysis

Statistical analysis was conducted via GraphPad Prism 8 with Fisher’s exact test, where the significance level was established at *p* < 0.05. To offer a succinct and transparent visualization of intricate AMR and virulence data relationships, an UpSetR plot was generated using R software version 4.3.2.

## Results

The study detected TYM and HIS in dairy items like Romy Cheese, Cheddar Cheese, Labn Rayeb, and Yoghurt (both small and large scale). Results were validated by a calibration curve and chromatogram (Figure S1). TYM and HIS were detected in 86.7% and 87.9% of samples, respectively. TYM concentrations were highest in Romy Cheese (693 mg/kg), followed by Cheddar Cheese (509 mg/kg), Labn Rayeb (Small scale) (425 mg/kg), and lower in Labn Rayeb large (Large scale) and Yoghurt samples (Small and Large scale) (26.1, 37.8 and 20.6 mg/kg, respectively). While HIS levels in dairy products: highest in Romy Cheese (725 mg/kg) and Cheddar Cheese (649 mg/kg); Labn Rayeb (Small scale) (425 mg/kg); and lowest in Yoghurt (Small scale) (29.5 mg/kg), Labn Rayeb (Large scale) (28.9 mg/kg), and Yoghurt (Large scale) (25.6 mg/kg) (Table 1).

**Table 1:** Incidence and levels of TYM and HIS (mg/kg) in the examined samples

Samples (No.)	TYM				HIS			
	Positive samples No. (%)	Min.	Max.	Mean	Positive samples No. (%)	Min.	Max.	Mean
Romy (Ras) Cheese (40)	40 (100)	84	1280	693	40 (100)	197	1600	725
Cheddar Cheese (40)	40 (100)	174	948	509	40 (100)	190	1050	649
Labn Rayeb - Small scale (40)	37 (93)	116	794	425	36 (90)	265	860	425
Labn Rayeb - Large scale (40)	29 (73)	12.9	57.9	26.1	31 (78)	11.2	65	28.9
Yoghurt - Small scale (40)	33 (83)	14.7	89.1	37.8	35 (88)	13.9	77.6	29.5
Yoghurt - Large scale (40)	29 (73)	13.6	46.3	20.6	29 (73)	22.4	56	25.6
Total (240)	208 (86.7)	-	-	285.3	211 (87.9)	-	-	313.8

Table S3, shows biogenic amine (BA) levels exceeding the Maximum Permissible Limit (MPL) in various dairy samples: Romy (Ras) Cheese, Cheddar Cheese and Labn Rayeb (Small scale): 100% of samples exceeded MPL for TYM and HIS; Labn Rayeb (Large scale): 7% exceed MPL for TYM and 0% for HIS. While 39% of Yoghurt (Small scale) exceeded MPL for TYM and 0% for HIS; moreover, all Yoghurt (Large scale) samples were within the MPL. Furthermore, Tables S4 and S5 provide a comparative analysis of Acceptable Intake (AI) vs. Estimated Intake (EI) of TYM and HIS in various food samples for children and adults. Both children and adults exceed the AI and PTWI for TYM, with children exceeding 80% and adults by 35% in Romy (Ras) Cheese. In Cheddar, both groups exceed the AI and PTWI, with children exceeding by 70% and adults by 7.5%, while high levels of TYM exceed AI and PTWI for children (67.5%) but not for adults in Labn Rayeb (Small scale). Both children and adults are within safe limits for TYM in Labn Rayeb (Large scale) and Yoghurt (Small and Large scale). In regards oh HIS, both children and adults exceed the AI and PTWI for histamine, indicating high exposure risk in Romy (Ras) Cheese samples. Similar to Romy Cheese, both groups exceed the AI and PTWI, suggesting significant histamine presence in Cheddar Cheese. In Labn Rayeb (Small scale): High levels of histamine exceed AI and PTWI for children and adults (90%). Histamine levels are within safe limits for adults in Labn Rayeb and Yoghurt (Large scale).

Table 2 shows that 52.9% of ready-to-eat dairy products had *Enterococcus* bacteria: *E. faecalis* (27.9%), *E. faecium* (24.2%), and *E. casseliflavus* (0.8%). All bacterial strains of *Enterococcus* spp. that were isolated from the samples, in the current study, underwent investigation for their tyrosine and histidine decarboxylase activity. Decarboxylase microplate assay revealed that 31.5% of *E. faecium* and *E. faecalis* strains could form BAs, but *E. casseliflavus* could not. Of 40 enterococci isolates, 85% had *tyrdc* and 5% had *hdc* amplicons (Table 3).

**Table 2:** Incidence of *Enterococcus* spp. In the examined samples

Samples	No. of samples	Positive samples for the presence of *Enterococcus* spp.		*E. faecalis*		*E. faecium*		*E. casseliflavus*	
		No.	%	No.	%	No.	%	No.	%
**Romy (Ras) Cheese**	40	30	75	25	62.5	4	10	1	2.5
**Cheddar Cheese**	40	26	65	9	22.5	17	42.5	0	0
**Labn Rayeb – Small scale**	40	27	67.5	16	40	10	25	1	2.5
**Labn Rayeb-Large scale**	40	5	12.5	0	0	5	12.5	0	0
**Yoghurt - Small scale**	40	35	87.5	13	32.5	22	55	0	0
**Yoghurt - Large scale**	40	4	10	4	10	0	0	0	0
**Total**	240	127	52.9	67	27.9	58	24.2	2	0.8

**Table 3:** Phenotypic and genotypic determination of tyrosine and histidine decarboxylation in *Enterococcus isolates*

Isolates	^a^No. of Isolates	Phenotypic detection of TYM and HIS		Genotypic detection of tyrosine and histidine decarboxylation encoding genes			
				*tyrdc*		*hdc*	
		No.	%	No.	%	No.	%
***Enterococcus spp.***	127	40^b^*	31.5	34^c, d*^	85	2	5

^a^ number of *Enterococcus spp.* isolated from the examined samples

^b^ significant correlation between amino acid decarboxylase activity on decarboxylase microplate assay and HPLC results

^c^ significant correlation between *tyrdc* positive isolates and TYM detection by HPLC

^d^ significant correlation between *tyrdc* positive isolates and amino acid decarboxylase activity on decarboxylase microplate assay

^*^ Statistically significant (*P* < 0.05)

**Fig. 1:**
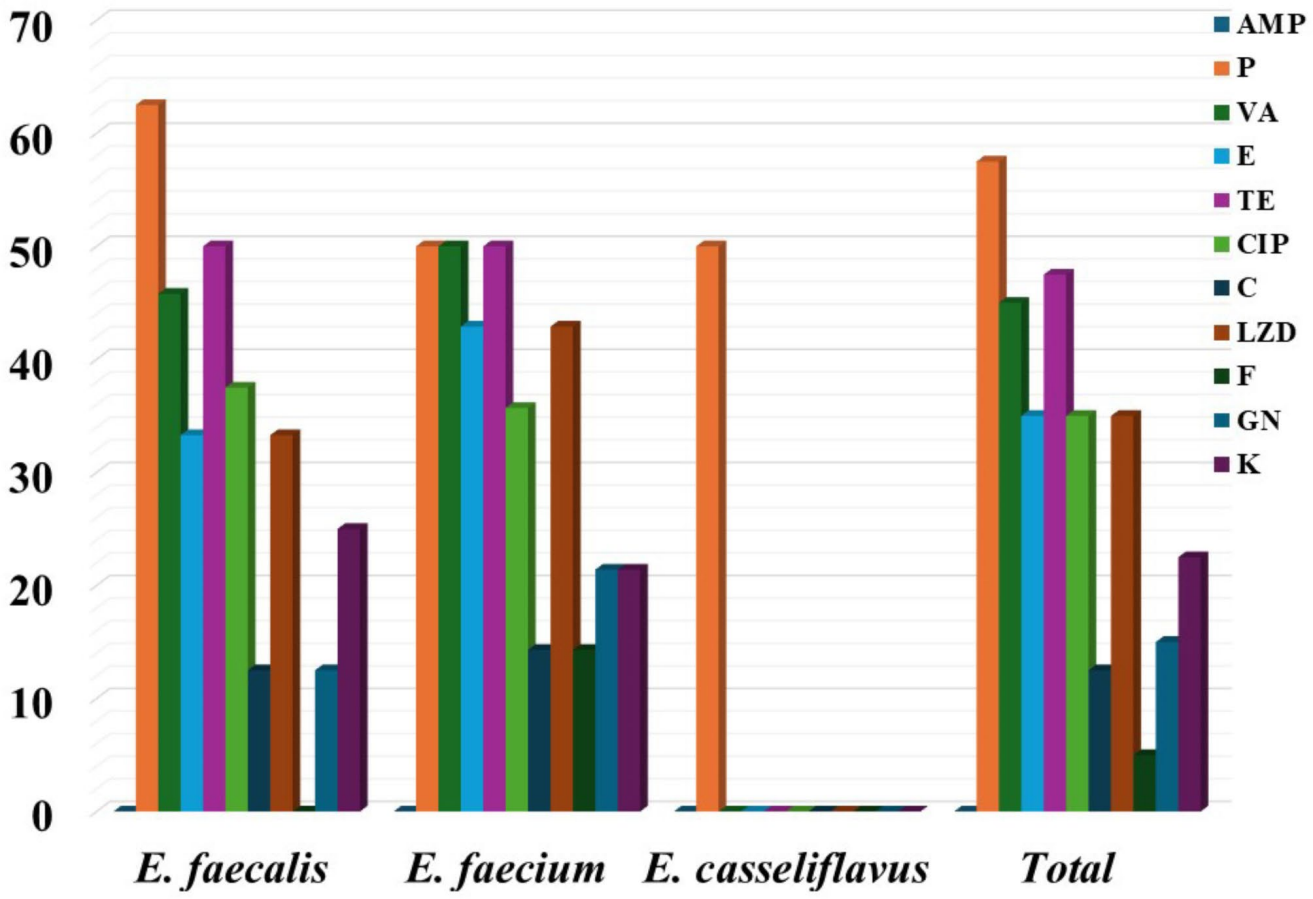
Antimicrobial resistance of tyrosine and histidine decarboxylating *Enterococcus* spp. Ampicillin (AMP); Penicillin (P); Vancomycin (VA); Erythromycin (E); Tetracycline (TE); Ciprofloxacin (CIP); Chloramphenicol (C); Linezolid (LZD); Nitrofurantoin (F); Gentamicin (CN); Kanamycin (K)

**Fig. 2:**
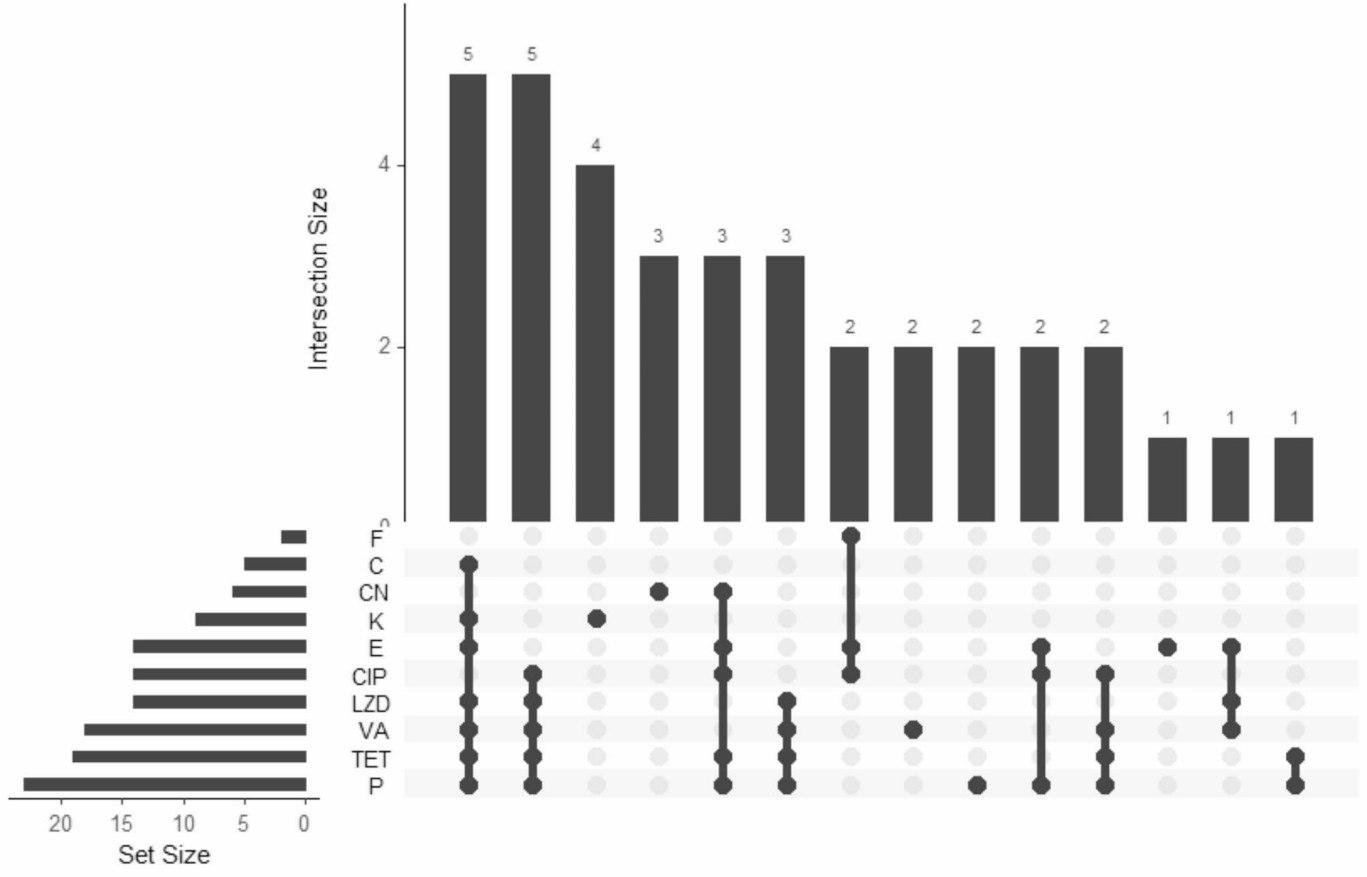
Antimicrobial resistance patterns of tyrosine and histidine decarboxylating *Enterococcus* spp. Penicillin (P); Vancomycin (VA); Erythromycin (E); Tetracycline (TE); Ciprofloxacin (CIP); Chloramphenicol (C); Linezolid (LZD); Nitrofurantoin (F); Gentamicin (CN); Kanamycin (K)

**Fig. 3:**
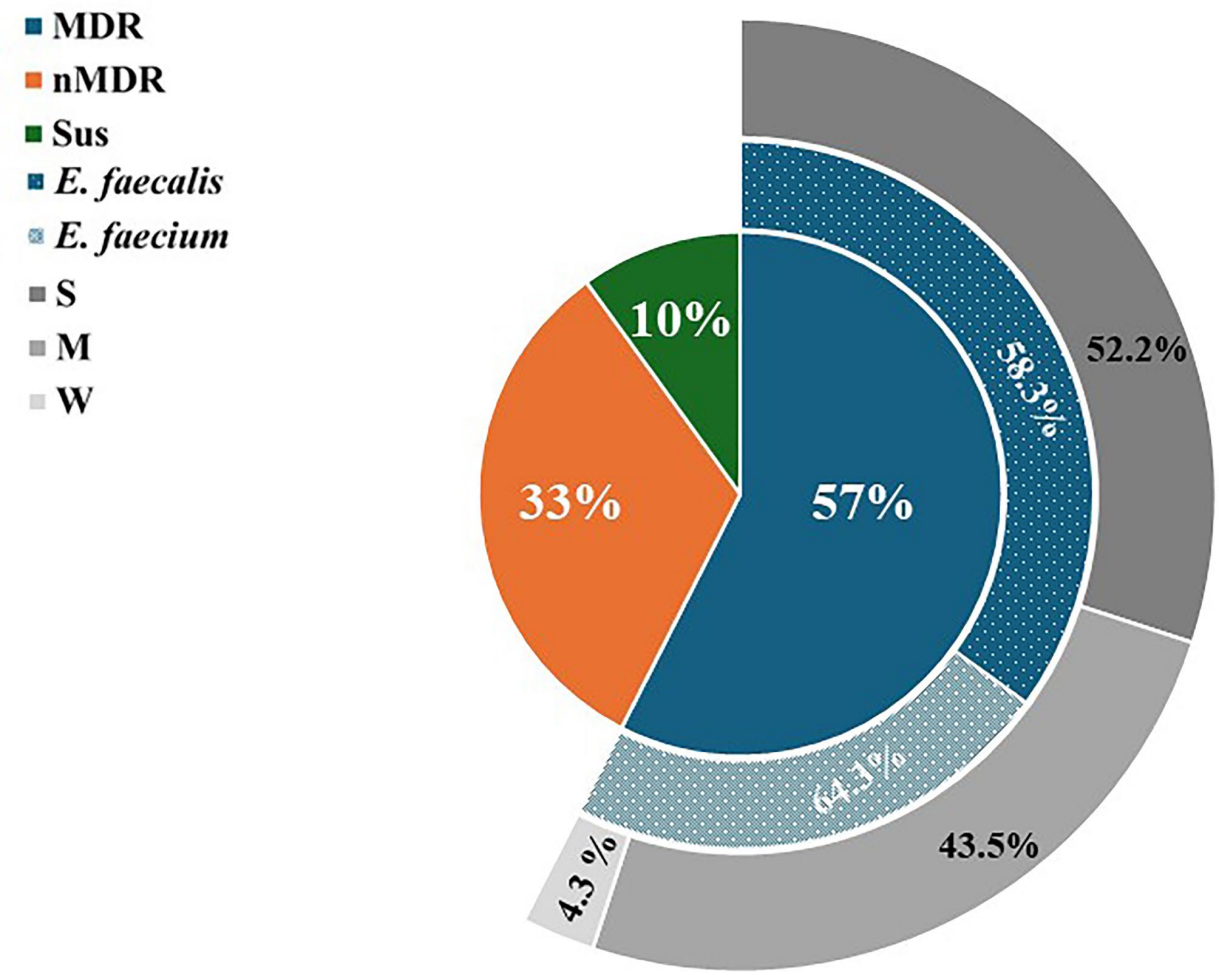
Drug resistance profile of tyrosine and histidine decarboxylating *Enterococcus* isolates concerning biofilm production. MDR: multi-drug-resistant strains; nMDR: isolates show resistance to less than 3 antimicrobials of different groups; Sus: Antimicrobial susceptible strains; S: strong biofilm producers; M: moderate biofilm producers; W: weak biofilm producers

Antimicrobial resistance testing of 40 *Enterococcus* isolates showed 90% were resistant to at least one antibiotic. Penicillin G had the highest resistance (57.5%), followed by tetracycline (47.5%) and vancomycin (45%). Additionally, a notable number of isolates showed resistance to Linezolid, erythromycin, ciprofloxacin, kanamycin, gentamycin, chloramphenicol, and Nitrofurantoin, albeit at different percentages (Fig. 1). Fourteen resistance patterns were identified; 58.3% of *E. faecalis* and 64.3% of *E. faecium* were multidrug-resistant (MDR). Overall, 57.5% of isolates were MDR (Table S6, Figs. 2 and 3).

This study found that 77.5% of *Enterococcus* isolates formed biofilms: 22.5% non-formers, 20% weak, 27.5% moderate, and 30% strong formers (Table 4). Biofilm formation was significantly linked to drug resistance (*p* < 0.0001). Among MDR isolates, 52.2% were strong, 43.5% moderate, and 4.3% were weak biofilm producers (Fig. 3).

**Table 4:** Biofilm formation by tyrosine and histidine decarboxylating *Enterococcus* spp

Biofilm	*Enterococcus* spp. (No.)			
	*E. faecalis *(24)	*E. faecium* (14)	*E. casseliflavus* (2)	Total
	No. (%)	No. (%)	No. (%)	No. (%)
**Strong**	8 (33.3)	4 (28.6)	0 (0)	12 (30)
**Moderate**	7 (29.2)	4 (28.6)	0 (0)	11 (27.5)
**Weak**	3 (12.5)	4 (28.6)	1 (50)	8 (20)
**Non-Biofilm producer**	6 (25)	2 (14.3)	1 (50)	9 (22.5)

**Fig. 4:**
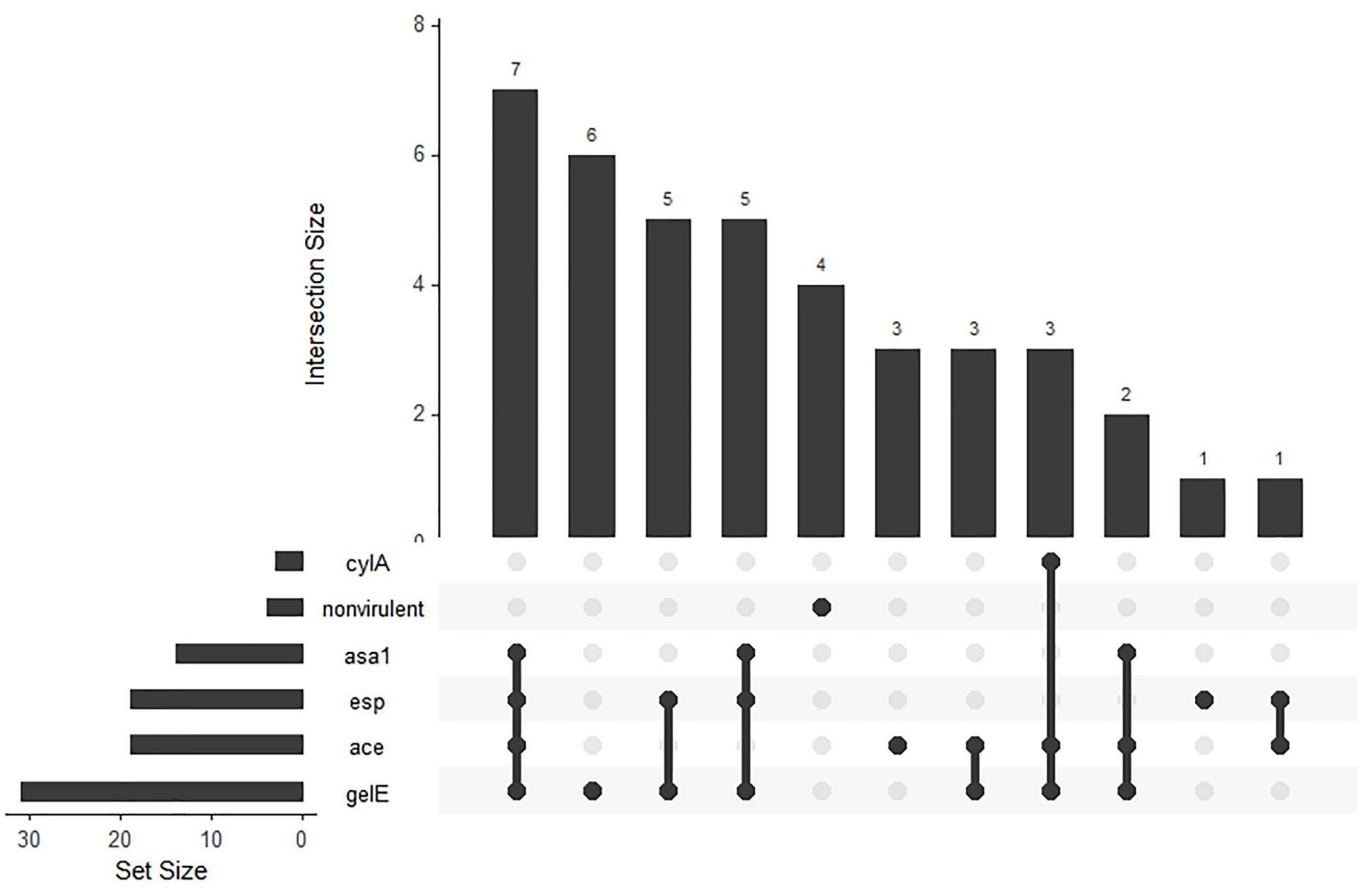
Virulence profile of tyrosine and histidine decarboxylating *Enterococcus* spp

**Fig. 5:**
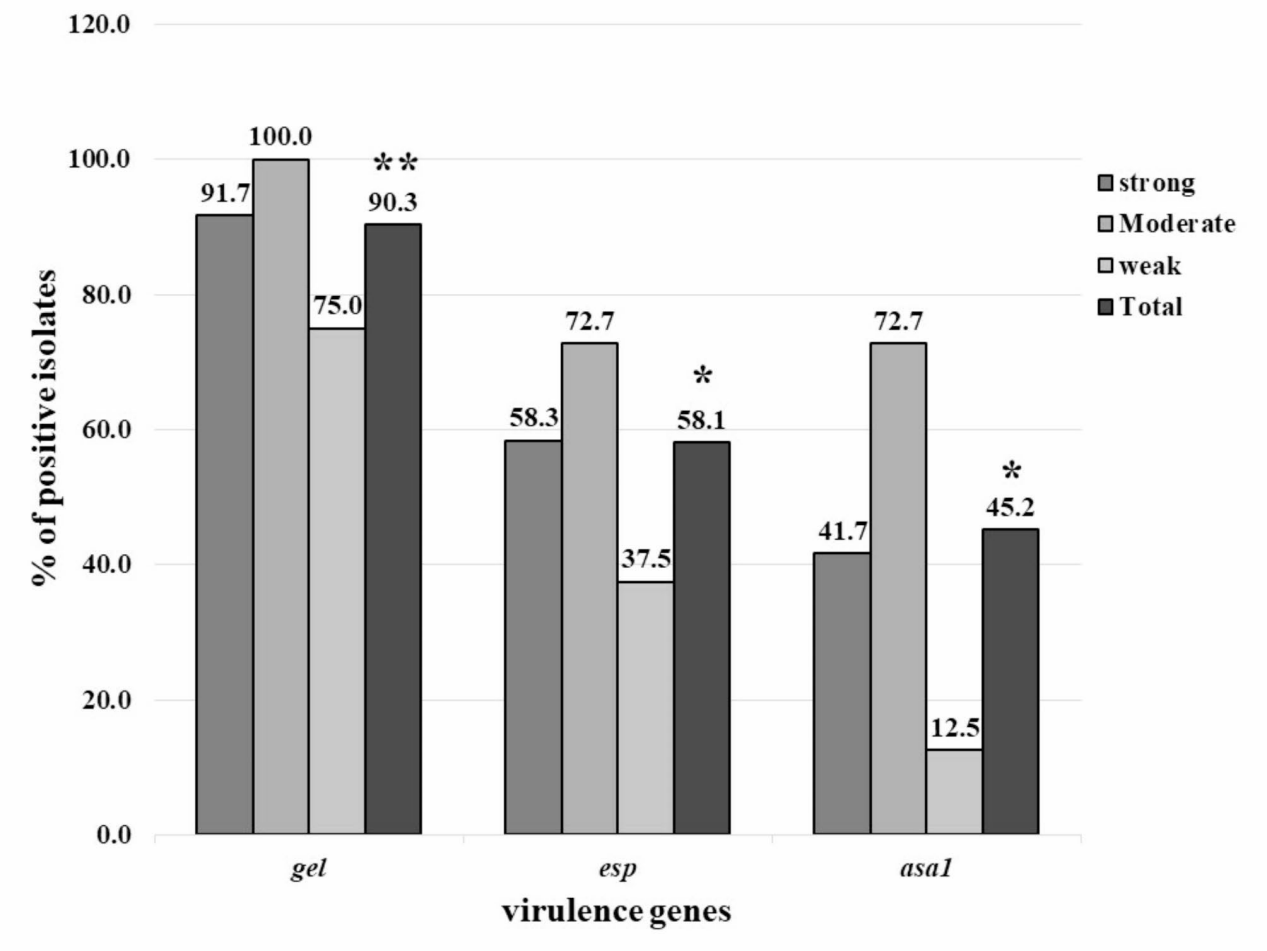
Relationship between the prevalence of virulence genes and biofilm formation ability of tyrosine and histidine decarboxylating *Enterococcus* spp. isolates. ** *P* = 0.001; * (*P* < 0.05)

Enterococci isolates had six virulence genes; *gelE* was most prevalent (77.5%), followed by *esp* and *ace* (47.5%), *asa1* (35.0%), and *cylA* (7.5%). No *hyl* gene was detected (Table 5). Furthermore, 90% of isolates had at least two virulence genes. Ten virulence profiles were identified, with *gelE* and *ace* being the most common (Fig. 4). A positive correlation was found between *esp* and *asa1* genes and biofilm formation (Fig. 5).

**Table 5:** Virulence determinants in tyrosine and histidine decarboxylating *Enterococcus* spp

Enterococcus spp. (No.)	Virulence determinants					
	*ace*	*cylA*	*gelE*	*hyl*	*esp*	*asa1*
	No. (%)	No. (%)	No. (%)	No. (%)	No. (%)	No. (%)
***E. faecalis (*** **24)**	11 (45.8)	3 (12.5)	21 (87.5)	0 (0)	10 (41.7)	9 (37.5)
***E. faecium*** **(14)**	8 (57.1)	0 (0)	10 (71.4)	0 (0)	9 (64.3)	5 (35.7)
**Total (40)**	19 (47.5)	3 (7.5)	31 (77.5)	0 (0)	19 (47.5)	14 (35)

**Fig. 6:**
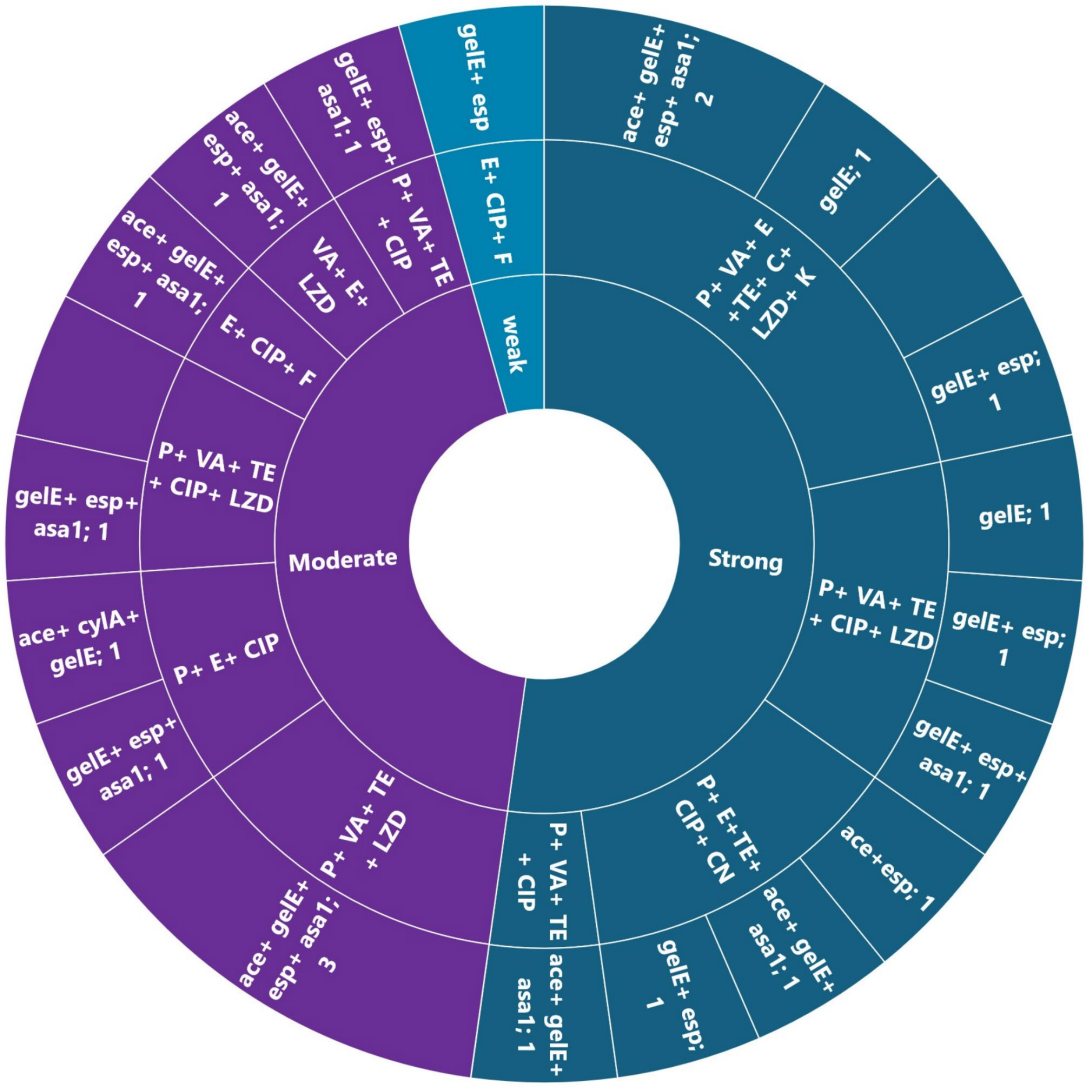
Biofilm production, AMR and virulence profiles in tyrosine and histidine decarboxylating MDR *Enterococcus* spp. isolates. Ampicillin (AMP); Penicillin (P); Vancomycin (VA); Erythromycin (E); Tetracycline (TE); Ciprofloxacin (CIP); Chloramphenicol (C); Linezolid (LZD); Nitrofurantoin (F); Gentamicin (CN); Kanamycin (K)

Figure 6 shows all MDR *Enterococcus* isolates had virulence genes. The most common profile (*ace* + *gelE* + *esp* + *asa1*) was in 21.7% of isolates. Among these, two were resistant to 7 antimicrobials (MAR index 0.64), and three to 4 antimicrobials (MAR index 0.36) (Table S6). A significant correlation was found between the *esp* gene and vancomycin resistance (*p* < 0.05).

## Discussion

Biogenic amines (BAs) indicate food spoilage and health risks when present in high concentrations, varying by dairy product type, ripening duration, and microflora activity, particularly in terms of decarboxylation. TYM levels were reported lower by Swelam and Mehanna (12.2 mg/kg) [31] and Farag et al. (202.7 mg/kg) [32] in Romy Cheese; Elkassas and El-bahy [33] (145.1 mg/kg) and Kandasamy et al. [34] (208.39 mg/kg) in Cheddar Cheese; Kandasamy et al. [34] (209.2 mg/kg) in Fermented milk. Similar results were found by Vallejos et al. [35] (571.3 mg/kg) in Cheddar Cheese, while Costa et al. [36] reported higher levels (560 mg/kg) in Fermented milk. TYM is the most common biogenic amine in dairy products that can exceed 1000 mg/kg in cheese and is frequently associated with biogenic amine-mediated intoxications [37]. Consuming 20–100 mg of TYM can cause toxic effects, with 100–800 mg/kg being hazardous, leading to ‘cheese reaction [38, 39]. TYM contamination poses risks like hypertension, nausea, headaches, emesis, respiratory discomfort, rashes, and heart palpitations [40]. Awareness of these risks is crucial.

In regards to HIS, Swelam and Mehanna [31] reported the highest HIS levels in Romy Cheese (1149 mg/kg), followed by Kandasamy et al. [34] (1468.46 mg/kg). Lower concentrations were found by Nazem et al. [41] (84.9 mg/kg), Farag et al. [32] (150 mg/kg), and El-Kholy et al. [42] (126.77 mg/kg). In Cheddar Cheese, El-Kholy et al. [42] reported 9.96 mg/kg, and Kandasamy et al. [34] found 176.8 mg/kg. El-Leboudy [43] et al. reported 77 mg/kg in Fermented milk, and Abo El-Makarem and Amer [44] found 645 mg/kg in Romy Cheese. These findings highlight HIS variability in dairy products and the need for monitoring and reporting such concentrations for food safety and quality purposes.

TYM and HIS levels in dairy products often exceeded international limits. According to FDA and Egyptian standards, 26.67%, 13.33%, and 20% of Cheddar Cheese samples, and 20%, 20%, and 6.67% of Romy Cheese samples exceeded limits for TYM or HIS, respectively in the study of Ibrahim and Amer [45]. However, Abd El-Salam et al. [46] and Aladhadh et al. [47] found all cheese samples within permissible limits established by the US Food and Drug Administration [48] and the Egyptian Organization for Standardization [49], highlighting discrepancies in the results compared to standards set by different regulatory bodies and the need for consistent and standardized regulations to ensure the safety and quality of dairy products for consumers. Our results also emphasize the significant presence of BAs in certain dairy products, particularly those produced on a small scale, and underscores the importance of monitoring and controlling BA levels to ensure food safety.

Elevated BAs in Ras cheese may result from microbial proteases, amino acids, substandard materials, contamination, poor processing, and storage [44]. Fermentation (25–44 °C), maturation (10–20 °C) and elevated storage temperatures [50, 51], pH (5.0-6.5), water activity (0.90-1.00) [52], and salt content (0.5–2 g/100 g) [53] all contribute to BA formation. High occurrences of BAs in this study are due to these factors. Significant variations in amine levels have been noted even among products of the same kind. These variations are influenced by microflora, chemical and physical variables, hygiene protocols, and the presence of precursors [54].

The evaluation of health risks from BAs in dairy products compared ADI and PTWI with EDI and EWI levels for children and adults. The study also looked at AI and EI of BAs based on consumption frequency. Overall, the results indicate that Romy (Ras) Cheese, Cheddar Cheese, and small-scale production of Labn Rayeb has higher incidences of TYM and HIS exceeding safety thresholds compared to large-scale production and Yoghurt samples underscoring the need for careful monitoring of TYM and HIS levels in certain dairy products, especially those produced on a small scale, to ensure consumer safety. Age can complicate the issue of biogenic amines in several ways: (1) Metabolism: As we age, our body’s ability to metabolize and eliminate biogenic amines can slow down, leading to higher levels of these compounds in the body [47]; (2) Diet: Older adults may have dietary restrictions or changes that affect their intake of biogenic amines [55]; (3) Medications: Many older adults take medications that can interact with biogenic amines, either increasing their levels or affecting their metabolism [56] and (4) Health Conditions: Age-related health conditions, such as liver or kidney disease, can impair the body’s ability to process biogenic amines, leading to potential health issues [57].

Histamine and tyramine cause toxic effects by interacting with various receptors and enzymes. Histamine binds to four types of receptors (H1, H2, H3, H4) [58]. Activation of H1 receptors causes vasodilation, increased vascular permeability, bronchoconstriction, and itching. H2 receptor activation stimulates gastric acid secretion and increases heart rate, H3 and H4 receptors are primarily involved in neurotransmission and immune modulation [59]. In light of the above, histamine toxicological effects include headaches, sweating, burning nasal secretion, facial flushing, bright red rashes, dizziness, itching rashes, oedema (eyelids), urticaria, difficulty in swallowing, diarrhoea, respiratory distress, bronchospasm, increased cardiac output, tachycardia, extrasystoles, blood pressure disorders [60]. While TYM displaces norepinephrine in neurons, causing its sudden release and excessive adrenergic stimulation [61]. This leads to symptoms like headaches, migraine, neurological disorders, nausea, vomiting, respiratory disorders, hypertension, and risk of intracranial haemorrhage or stroke due to extreme hypertension [60], especially in individuals with impaired Monoamine Oxidase (MAO) activity [62].

Elevated BAs indicate unsanitary production and storage conditions. This research focused on the presence of enterococci and its involvement in the production of BAs via the decarboxylation of amino acids. Enterococci, prevalent in Mediterranean artisan dairy products, thrive in harsh conditions, as well as their adaptability, contributes to their widespread presence in foods [63].

*E. faecalis* is the most prevalent species at 27.9%, followed by *E. faecium* at 24.2%. This aligns with studies by Anderegg et al. [64], El-Zamkan and Mohamed [22], Gołaś-Prądzyńska et al. [65], Gajewska et al. [66], Morandi et al. [12], and Wang et al. [11], but Chajęcka-Wierzchowska et al. [67] found higher *E. faecium* prevalence. In line with the current findings, Terzić-Vidojević et al. [63] also noted *E. faecium* and *E. faecalis* as common in raw-milk cheeses. Many studies have linked *Enterococcus* spp. to biogenic amine (BA) formation in fermented dairy products [43, 67–70]. This study found a significant link between enterococci presence and BA production (*P* < 0.0001). Specific strains of *E. faecalis* and *E. faecium* produce TYM and HIS [64, 71, 72], highlighting the role of enterococci in biogenic amine production, which is important for food safety and quality.

In respect of the severity of symptoms they may cause, EFSA [4] identified HIS and TYM as major toxicological amines as HIS is associated with “scombroid fish poisoning” while TYR is linked to the “cheese reaction.” [2]. In this study, 31.5% of *Enterococcus* strains (*E. faecium*, *E. faecalis*) produced BAs via decarboxylase activity. Higher results were reported by Ismail [73] who found all *E. faecalis* and *E. faecium* in fermented milk could form TYM. Kalhotka [74] found all enterococci decarboxylated tyrosine but not histamine. Espinosa-Pesqueira [6] confirmed *E. faecalis* and *E. faecium* as the main amine producers in cheese. The microplate assay was effective for identifying low decarboxylase activity and processing many samples simultaneously, increasing the efficiency with low false positives and false negatives and enhancing screening reliability [6]. Findings align with various studies on *Enterococcus* spp. as prevalent TYM formers in food [64, 72, 75].

Molecular methods offer a fast, accurate alternative to culture-based identification of BA-producing bacteria by targeting genes encoding amino acid decarboxylase enzymes. PCR was used to detect *tyrdc* and *hdc* genes in enterococci isolates which amplified in 85and 5% of the isolates, respectively. Similar findings were reported by Komprda [76, 77], Bhardwaj [78], and Kalhotka [74], indicating a high prevalence of *tyrdc* and *tdc* genes in enterococci, with *hdc* being less common.

The study found a significant correlation between amino acid decarboxylase activity (measured by microplate assay) and HPLC analysis (*P* < 0.0001). Also, *tyrdc*-positive isolates correlated significantly with HPLC TYM detection and decarboxylase activity (*P* < 0.0001), but no significant relationship was found with the *hdc* gene. This aligns with studies by Komprda [76], Bhardwaj [78], and Espinosa-Pesqueira [6], which also found strong correlations. Six *Enterococcus* isolates lacked *tyrdc* and *hdc* genes but showed positive decarboxylase activity. This percentage is significantly lower compared to the findings of Roig-Sagués et al. [79, 80] and Hernández-Herrero et al. [81]. Discrepancies suggest possible false positives or alternative gene sequences [82]. Thorough validation and confirmation of decarboxylase activity is crucial to avoid misinterpretation.

Only two isolates were confirmed as histamine producers (*hdc* gene). Komprda et al. [76] reported a higher incidence. However, it is essential to consider other histidine decarboxylating bacteria may also contribute to high HIS levels, as previous studies noted [82, 83].

Discrepancies in decarboxylase activity confirmation rates highlight the complexity of bacterial enzymatic capabilities and the importance of molecular techniques. These techniques, like PCR, can identify BA-producing bacteria before BAs are detectable, helping estimate potential BA accumulation in the final product. PCR has been used in milk curd and cheese analysis to identify TYM-producing bacteria during cheese processing [37, 84], benefiting dairy manufacturers at any stage. Furthermore, it showed a strong correlation with HPLC results in commercially available cheese samples [85].

Antimicrobial resistance (AMR) in the food chain is a significant public health concern. Antimicrobial resistance testing of the 40 *Enterococcus* isolates showed 90% resistance to at least one antibiotic which is a higher resistance rate compared to previous studies by Terzić-Vidojević et al. [63] and Paschoalini et al. [86]. Our study reaffirmed resistance to penicillin, vancomycin, linezolid, erythromycin, and Nitrofurantoin with lower resistance levels compared to El-Zamkan and Mohamed [22].

Penicillin resistance in our study’s isolates is due to overexpression of low-affinity Penicillin-binding proteins in *Enterococcus* spp causing bacteria to be intrinsically resistant to penicillin. Our findings show higher resistance to vancomycin and erythromycin in *E. faecium* compared to Salamandane et al. [87]. Igbinosa and Beshiru [88] reported higher penicillin resistance and lower vancomycin resistance in *Enterococcus* spp. The dairy environment has extensive tetracycline and erythromycin resistance [89]. These patterns highlight the evolving nature of antibiotic resistance in *Enterococcus* spp. and the need for continuous monitoring.

A high prevalence of MDR was observed in *Enterococcus* isolates (57.5%). Gołaś-Prądzyńska et al. [65] and Morandi et al. [12] reported lower incidences of MDR, while El-Zamkan and Mohamed [22] reported a higher percentage of MDR among *Enterococcus* isolates. MDR was more prevalent in *E. faecium* than in *E. faecalis*, contradicting some previous studies [65, 87]. Common resistant antibiotics included penicillin, vancomycin, tetracycline, and linezolid, aligning with other studies [22, 90].

The WHO classifies vancomycin-resistant enterococci (VRE) as a high-priority pathogen, highlighting the need for effective strategies [16]. In this study vancomycin resistance was in five out of eight MDR patterns, with all VRE also resistant to linezolid. Linezolid-resistant VRE is a concern, given its importance in treating MDR Gram-positive bacteria since its introduction in the early 2000s [15]. The study confirmed the increasing trend of linezolid-resistant enterococci as previously described by Zarzecka et al. [18]. High MDR enterococci recovery indicates a significant reservoir of antibiotic resistance. Overuse of antibiotics in veterinary practices contributes to this issue [91], emphasizing the need for coordinated global action to combat antibiotic resistance. Understanding the mechanisms of resistance and monitoring resistance patterns is crucial for developing effective strategies to preserve the efficacy of existing antibiotics and prevent the spread of resistant bacteria.

Inadequate cleaning and disinfection promote bacterial biofilm development, representing a continuous source of contamination with pathogenic microorganisms. Igbinosa and Beshiru [88] and Wang et al. [11] recorded lower percentages, while Salamandane et al. [87] reported higher ones. There is a variance in biofilm production among *Enterococcus* species, with some studies showing higher occurrence in *E. faecalis* [29, 92] and others in *E. faecium* [22, 88]. However, this study found no significant difference (*p* > 0.05) in biofilm formation between *E. faecalis* and *E. faecium*, consistent with Gajewska et al. [66]. The relationship between drug resistance and biofilm formation was statistically significant (*P* < 0.0001). In the food production environment, biofilms play a crucial role in transferring resistance factors [88].

Enterococci are known for their virulence factors that contribute to their pathogenicity. Varied detection rates of virulence genes were documented. *GelE* was most prevalent in this and other studies Wang et al. [11], Gajewska et al. [66] and Salamandane et al. [87]. Conversely, *esp* was found to be the most common gene in *Enterococcus* isolates by Igbinosa and Beshiru [88] and Morandi et al. [12] and the latter observed that *E. faecium* lacked virulence genes. In contrast to the current findings, *cylA* gene was undetected in several studies Wang et al. [11], Gajewska et al. [66], Salamandane et al. [87], Igbinosa and Beshiru [88] and Kim et al. [93] but Gołaś-Prądzyńska et al. [65] reported the presence of the *cylA* gene in *E. faecalis* and *E. faecium* strains at 14.3% and 5.5%, respectively, and Salamandane et al. [87] also noted a higher incidence of the *esp* gene. Wang et al. [11] reported the absence of the *hyl* gene in *Enterococcus* isolates, aligning with our findings, likely due to its association with clinical infections rather than environmental strains [94].

The high prevalence of *gelE* highlights its significance for public health. Gelatinase, regulated by *gelE*, is a crucial virulence factor for *Enterococcus* spp., aiding tissue invasion, immune evasion, biofilm formation, and host cell modulation enhancing the pathogenicity of *Enterococcus* and facilitating the establishment of persistent infections. *Esp* is also a key virulence factor for *E. faecalis* and *E. faecium*, contributing to the severity of infections observed in clinical settings [87].

Previous literature considered *gelE* unrelated to biofilm formation [66, 88, 95]. However, this study found a significant role for *gelE* in biofilm formation (90.3% of biofilm-producing isolates are *esp* positive) indicating its significant role in the biofilm formation process. A positive correlation between *esp* and *asa1* genes and biofilm formation was also found. These findings align with other studies [11, 22, 66, 87, 96], contradicting Chajęcka-Wierzchowska et al. [95] and Igbinosa and Beshiru [88] whose work suggested that the presence of the *esp* element was not associated with biofilm formation.

In the current study, all MDR *Enterococcus* isolates possess virulence genes. The most common profile, “*ace* + *gelE* + *esp* + *asa1*,” was found in 21.7% (5/23) of MDR isolates. Two isolates showed resistance to 7 antimicrobials (MAR index 0.64) and three to 4 antimicrobials (MRA index 0.36). Most virulent MDR isolates also resisted vancomycin, highlighting the relationship between virulence genes, antimicrobial resistance, and biofilm formation. Indeed, previous studies have highlighted vancomycin-resistant enterococci as potent biofilm producers [97, 98]. Also, a significant correlation (*p* < 0.05) was found between the *esp* gene and vancomycin resistance. This contributed to that the *esp* gene encodes proteins that modulate antibiotic release and enhance antibiotic resistance through the transfer of genetic determinants in biofilm [99–101]. It is also linked to resistance against imipenem, ampicillin, and ciprofloxacin [102]. Aligning with this study, Ochoa et al. [103] confirmed the connection between vancomycin resistance and *esp* presence, as they observed that 83.3% of VRE strains in healthcare settings were *esp* positive, similar to this study’s 72.2% (13/19).

The abundance of virulence factors, antimicrobial resistance, and BAs production makes *Enterococcus* spp. significant opportunistic pathogens in healthcare-associated infections. They are commonly found in fermented dairy products that can serve as a reservoir for both antimicrobial resistance genes and virulence factors and facilitate the transfer of these genes to the human microbiota through the food chain, posing health risks. Awareness of these dangers, especially in unpasteurized milk or dairy products, is crucial to mitigate the spread of multidrug-resistant, virulent and BAs producer phenotypes and protect public health.

Finally, we acknowledge that although the sample selection focuses on a specific range of cheese and fermented milk products, it provides valuable insights that may not yet encompass the full diversity of such products. Additionally, while TYM and HIS were prioritized due to their significant relevance to the samples analyzed, exploring other biogenic amines and microbial species that may contribute to BA presents an opportunity for further research.

## Conclusion

In conclusion, the analysed samples exhibited elevated concentrations of TYM and HIS exceeding the safety thresholds especially in those products produced on a small scale, along with a high presence of TYM-producing *Enterococcus* spp. The prevalence of MDR among *Enterococcus* spp. and their capability to transfer AMR genes through dairy products should not be overlooked. The results also showed high existence of VRE, and virulence genes associated with the ability of biofilm formation, with most strains resistant to linezolid, a last-resort antibiotic for enterococcal infections. This poses a significant risk, highlighting the need to educate animal-origin food producers and veterinarians about the consequences of improper antimicrobial use to prevent the spread of resistant isolates, including MDR strains, to humans through the food chain. The study also found a high prevalence of biofilm production among the isolated *Enterococcus* strains, which contributes to the persistence of multidrug-resistant and virulent enterococci throughout the food chain, acting as a significant root of food contamination. The high prevalence of TYM-producing *Enterococcus* species presents a notable public health concern, especially with the high prevalence of multidrug-resistant, biofilm production, and virulence in BAs-producing *Enterococcus* spp. in dairy products. This emphasizes the importance of thorough cleaning of equipment and utensils used in dairy food production to mitigate contamination risks. These findings highlight the necessity for detailed characterization, continuous monitoring, and control measures of enterococci isolated from dairy food, given their potential as reservoirs of antibiotic resistance and virulence genes, as well as producers of biogenic amines. Moreover, using high-quality raw materials and optimal technological conditions is crucial in reducing BAs production and accumulation in fermented dairy products.

## Electronic supplementary material

Below is the link to the electronic supplementary material.

Supplementary Material 1

## Data Availability

All datasets generated or analyzed during this study are included in the manuscript.

## Abbreviations

BAs: Biogenic amines
TYM: Tyramine
HIS: Histamine
EDI: Estimated Daily Intakes
HPLC: High-performance liquid chromatography
EWI: Estimated Weekly Intakes
AMR: Antimicrobial resistance
MDR: Multi-drug resistant
MAR index: Multiple antibiotic resistance index
DCA: Decarboxylase control assay
NDA: Negative decarboxylase assay
PDA: Positive decarboxylase assay
