# Supplementary material for: Molecular characterization of histidine and tyrosine decarboxylating Enterococcus species isolated from some milk products
Source: BMC Microbiol. 2025 Apr 23;25:234. doi: 10.1186/s12866-025-03940-6 (PMC12016370; doi:10.1186/s12866-025-03940-6)
Supplement: Supplementary file 1 — Supplementary Material 1 [file 12866_2025_3940_MOESM1_ESM.docx]

**Table S1. HPLC conditions**

| Parameter | Optimized conditions |
| --- | --- |
| Column | C18 Waters Spherisorb ODS-2 (150 by 4.60 mm, particle diameter 3 µm) |
| Mobile Phase | Acetonitrile: ultrapure water (60:40) |
| Flow Rate | 1.2 ml/min |
| UV Detector | 254 nm. |
| Injection Volume | 20 µl |
| Column Temperature | Ambient |

**Table S2. Primers sequences of antimicrobial resistance genes, amplicon sizes and cycling conditions.**

| Target gene | Primers sequences | Amplified segment (bp) | Primary  denaturation | Amplification (35 cycles) | | | Final extension | Reference |
| --- | --- | --- | --- | --- | --- | --- | --- | --- |
|  |  |  |  | Secondary denaturati-on | Annealing | Extension |  |  |
| *E. faecalis* 16S rRNA | GTT TAT GCC GCA TGG CAT AAG AG | 310 | 94˚C  5 min | 94˚C  30 sec | 50˚C  30 sec. | 72˚C  30 sec | 72˚C  7 min. | [26] |
|  | CCG TCA GGG GAC GTT CAG |  |  |  |  |  |  |  |
| *E. faecium atpA* | CGG TTC ATA CGGAAT GGC ACA | 556 | 94˚C  5 min | 94˚C  30 sec | 50˚C  40 sec | 72˚C  45 sec | 72˚C  10 min. | [ 27] |
|  | AAG TTC ACG ATA AGC CAC GG |  |  |  |  |  |  |  |
| *tyrdc* | CAAA-TGGA-AGAA-GAAG- TAGG | 1100 | 94˚C  15 min. | 95˚C  45 sec. | 52˚C  45sec. | 72˚C  75 sec. | 72˚C  10 min. | [28] |
|  | ACAT-AGTC-AACC-ATRT-TGAA |  |  |  |  |  |  |  |
| *hdc* | GATG-GTAT-TGTT-TCKT-ATGA | 440 | 94˚C  15 min. | 95˚C  45 sec. | 52˚C  45 sec | 72˚C  75 sec | 72˚C  7 min. |  |
|  | CAAA-CACC-AGCA-TCTT-C |  |  |  |  |  |  |  |
| *ace* | GGAATGACCGAGAACGATGGC | 616 | 94˚C  5 min. | 94˚C  30 sec. | 58˚C  40 sec. | 72˚C  45 sec. | 72˚C  10 min. | [29] |
|  | GCTTGATGTTGGCCTGCTTCCG |  |  |  |  |  |  |  |
| *cylA* | ACTCGGGGATTGATAGGC | 688 | 94˚C  5 min. | 94˚C  30 sec. | 50˚C  40 sec. | 72˚C  45 sec. | 72˚C  10 min. | [30] |
|  | GCTGCTAAAGCTGCGCTT |  |  |  |  |  |  |  |
| *gelE* | TATGACAATGCTTTTTGGGAT | 213 | 94˚C  5 min. | 94˚C  30 sec. | 50˚C  30 sec. | 72˚C  30 sec. | 72˚C  7 min. |  |
|  | AGATGCACCCGAAATAATATA |  |  |  |  |  |  |  |
| *hyl* | ACAGAAGAGCTGCAGGAAATG | 276 | 94˚C  5 min. | 94˚C  30 sec. | 55˚C  30 sec. | 72˚C  30 sec. | 72˚C  7 min. |  |
|  | GACTGACGTCCAAGTTTCCAA |  |  |  |  |  |  |  |
| *Esp* | AGATTTCATCTTTGATTCTTGG | 510 | 94˚C  5 min. | 94˚C  30 sec. | 50˚C  40 sec. | 72˚C  45 sec. | 72˚C  10 min. |  |
|  | AATTGATTCTTTAGCATCTGG |  |  |  |  |  |  |  |
| *Asa1* | GCACGCTATTACGAACTATGA | 375 | 94˚C  5 min. | 94˚C  30 sec. | 50˚C  40 sec. | 72˚C  45 sec. | 72˚C  10 min. |  |
|  | TAAGAAAGAACATCACCACGA |  |  |  |  |  |  |  |


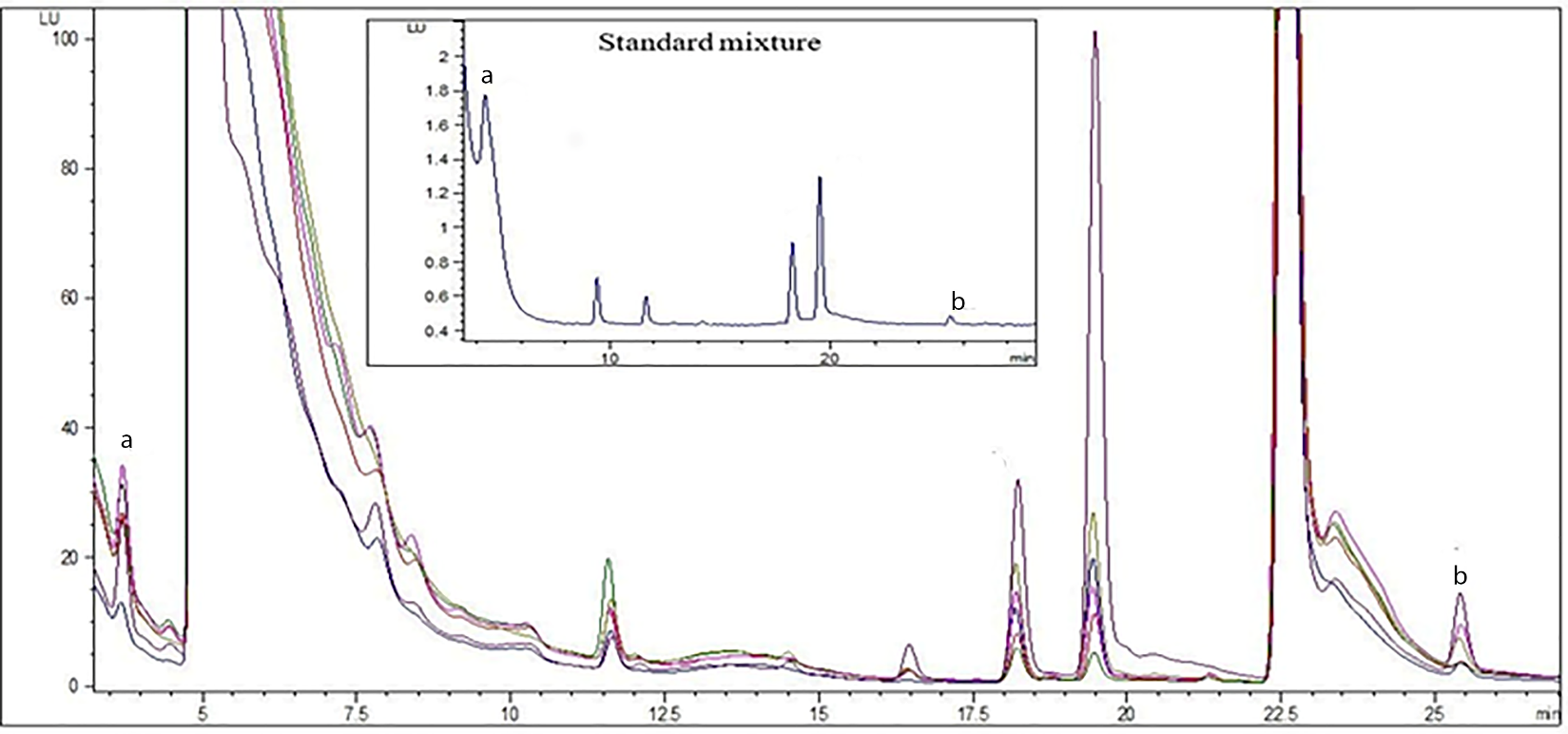


**Figure S1.** Samples representative chromatogram obtained from HPLC detection of biogenic amines; HIS (a) and TYM (b).

| **Biogenic amines** | **MPL**  **(mg/kg)** | **Romy (Ras) Cheese** | | **Cheddar Cheese** | | **Labn Rayeb (Fermented milk) - Small scale** | | **Labn Rayeb (Fermented milk) - Large scale** | | **Yoghurt - Small scale** | | **Yoghurt - Large scale** | |
| --- | --- | --- | --- | --- | --- | --- | --- | --- | --- | --- | --- | --- | --- |
|  |  | **>MPL** | | **>MPL** | | **>MPL** | | **>MPL** | | **>MPL** | | **>MPL** | |
|  |  | **No. of samples** | **%** | **No. of samples** | **%** | **No. of samples** | **%** | **No. of samples** | **%** | **No. of samples** | **%** | **No. of samples** | **%** |
| **TYM** | **50** | **40** | **100** | **40** | **100** | **37** | **100** | **2** | **7** | **13** | **39** | **0** | **0** |
| **HIS** | **100** | **40** | **100** | **40** | **100** | **36** | **100** | **0** | **0** | **0** | **0** | **0** | **0** |

**Table S3.** Incidence of biogenic amines levels exceeding the Maximum Permissible Limit (mg/kg) in the examined samples (N=40).

**Table S4. Comparative analysis of the AI with EI (mg/kg b.w.) of TYM from examined samples for children and adults.**

| **Biogenic amines** | **ADI** | **PT WI** | **Samples** | | **Children** | | | | **Adult** | | | |
| --- | --- | --- | --- | --- | --- | --- | --- | --- | --- | --- | --- | --- |
|  |  |  | **Type of sample** | **Mean conc. (mg/kg)** | **DI** | | **WI** | | **DI** | | **WI** | |
|  |  |  |  |  | **EDI** | **>ADI** | **EWI** | **>PTWI** | **EDI** | **>ADI** | **EWI** | **>PTWI** |
| **TYM** | **600^a^** | **4200** | **Romy (Ras) Cheese** | **693** | **1250** | **32 (80%)** | **8740** | **32 (80%)** | **480** | **14 (35%)** | **3360** | **14 (35%)** |
|  |  |  | **Cheddar Cheese** | **509** | **917** | **28 (70%)** | **6420** | **28 (70%)** | **353** | **3 (7.5%)** | **2470** | **3 (7.5%)** |
|  |  |  | **Labn Rayeb**  **- Small scale** | **425** | **764** | **27 (67.5%)** | **5350** | **27 (67.5%)** | **294** | **0 (0%)** | **2060** | **0 (0%)** |
|  |  |  | **Labn Rayeb**  **- Large scale** | **26.1** | **37.1** | **0 (0%)** | **259** | **0 (0%)** | **14.3** | **0 (0%)** | **99.8** | **0 (0%)** |
|  |  |  | **Yoghurt –**  **Small Scale** | **37.8** | **68** | **0 (0%)** | **476** | **0 (0%)** | **26.1** | **0 (0%)** | **183** | **0 s(0%)** |
|  |  |  | **Yoghurt – Large**  **scale** | **20.6** | **37.1** | **0 (0%)** | **259** | **0 (0%)** | **14.3** | **0 (0%)** | **99.8** | **0 (0%)** |

**^a^EFSA [4]**

**Table S5.** Comparative analysis of the AI with EI (mg/kg b.w.) of HIS from the examined samples for children and adults.

| **Biogenic amines** | **ADI** | **PTWI** | **Samples** | | **Children** | | | | **Adult** | | | |
| --- | --- | --- | --- | --- | --- | --- | --- | --- | --- | --- | --- | --- |
|  |  |  | **Type of sample** | **Mean conc. (mg/kg)** | **DI** | | **WI** | | **DI** | | **WI** | |
|  |  |  |  |  | **EDI** | **>ADI** | **EWI** | **>PTWI** | **EDI** | **>ADI** | **EWI** | **>PTWI** |
| **HIS** | **50^a^** | **350** | **Romy (Ras) Cheese** | **725** | **1310** | **40 (100%)** | **9140** | **40 (100%)** | **502** | **40 (100%)** | **3510** | **40 (100%)** |
|  |  |  | **Cheddar Cheese** | **649** | **1170** | **40 (100%)** | **8170** | **40 (100%)** | **449** | **40 (100%)** | **3140** | **40 (100%)** |
|  |  |  | **Labn Rayeb**  **- Small scale** | **425** | **764** | **36 (90%)** | **5350** | **36 (90%)** | **294** | **36 (90%)** | **2060** | **36 (90%)** |
|  |  |  | **Labn Rayeb**  **- Large scale** | **28.9** | **52** | **22 (55%)** | **364** | **22 (55%)** | **20** | **0 (0%)** | **140** | **0 (0%)** |
|  |  |  | **Yoghurt –**  **Small scale** | **29.5** | **53.1** | **20 (50%)** | **371** | **20 (50%)** | **20.4** | **1 (2.5%)** | **143** | **1 (2.5%)** |
|  |  |  | **Yoghurt –**  **Large scale** | **25.6** | **46** | **20 (50%)** | **322** | **20 (50%)** | **17.7** | **0 (0%)** | **124** | **0 (0%)** |

**^a^EFSA [4]**

Table S6. Antimicrobial resistance patterns of *Enterococcus* spp.

| No. of AMR | AMR pattern | *E. faecalis*  No. (%) | *E. faecium*  No. (%) | *E. casseliflavus*  No. (%) | Total  No. (%) | MAR Index |
| --- | --- | --- | --- | --- | --- | --- |
| 0 | 0 | 3 (7.5) | 0 (0) | 1 (2.5) | 4 (10) | 0.00 |
| 1 | GN | 1 (2.5) | 2 (5) | 0 (0) | 3 (7.5) | 0.09 |
|  | K | 3 (7.5) | 1 (2.5) | 0 (0) | 4 (10) |  |
|  | VA | 1 (2.5) | 1 (2.5) | 0 (0) | 2 (5) |  |
|  | P | 1 (2.5) | 0 (0) | 1 (2.5) | 2 (5) |  |
|  | E | 0 (0) | 1 (2.5) | 0 (0) | 1 (2.5) |  |
| 2 | P; TET | 1 (2.5) | 0 (0) | 0 (0) | 1 (2.5) | 0.02 |
| 3 | P, E, CIP | 2 (5) | 0 (0) | 0 (0) | 2 (5) | 0.27 |
|  | VA; E; LZD | 1 (2.5) | 0 (0) | 0 (0) | 1 (2.5) |  |
|  | E, CIP, NITR | 0 (0) | 2 (5) | 0 (0) | 2 (5) |  |
| 4 | P; VA; TE; LZD | 1 (2.5) | 2 (5) | 0 (0) | 3 (7.5) | 0.36 |
|  | P; VA; TE; CIP | 2 (5) | 0 (0) | 0 (0) | 2 (5) |  |
| 5 | P; E; TE; CIP; Gen | 2 (5) | 1 (2.5) | 0 (0) | 3 (7.5) | 0.45 |
|  | P; VA; TE; CIP; LZD | 3 (7.5) | 2 (5) | 0 (0) | 5 (12.5) |  |
| 7 | P; VA; E; TE; CHL; LZD; K | 3 (7.5) | 2 (5) | 0 (0) | 5 (12.5) | 0.64 |
| Average MAR Index | | | | |  | 0.26 |
